# Intraarticularly-Injected Mesenchymal Stem Cells Stimulate Anti-Inflammatory Molecules and Inhibit Pain Related Protein and Chondrolytic Enzymes in a Monoiodoacetate-Induced Rat Arthritis Model

**DOI:** 10.3390/ijms19010203

**Published:** 2018-01-09

**Authors:** Toru Ichiseki, Miyako Shimasaki, Yoshimichi Ueda, Shusuke Ueda, Masanobu Tsuchiya, Daisuke Souma, Ayumi Kaneuji, Norio Kawahara

**Affiliations:** 1 Department of Orthopaedic Surgery, Kanazawa Medical University, Daigaku 1-1, Uchinada, Kahoku-gun, Ishikawa 920-0293, Japan; adeu221@kanazawa-med.ac.jp (S.U.); m-tsv@kanazawa-med.ac.jp (M.T.); ds0924@kanazawa-med.ac.jp (D.S.); orthoped@kanazawa-med.ac.jp (A.K.); kawa@kanazawa-med.ac.jp (N.K.); 2 Department of Pathology, Kanazawa Medical University, Daigaku 1-1, Uchinada, Kahoku-gun, Ishikawa 920-0293, Japan; miya0807@kanazawa-med.ac.jp (M.S.); z-ueda@kanazawa-med.ac.jp (Y.U.)

**Keywords:** osteoarthritis, mesenchymal stem cell, central sensitization, adamts5, tnf-α stimulated gene/protein 6, calcitonin gene related peptide

## Abstract

Persistent inflammation is well known to promote the progression of arthropathy. mesenchymal stem cells (MSCs) have been shown to possess anti-inflammatory properties and tissue differentiation potency. Although the experience so far with the intraarticular administration of mesenchymal stem cell (MSC) to induce cartilage regeneration has been disappointing, MSC implantation is now being attempted using various surgical techniques. Meanwhile, prevention of osteoarthritis (OA) progression and pain control remain important components of the treatment of early-stage OA. We prepared a shoulder arthritis model by injecting monoiodoacetate (MIA) into a rat shoulder, and then investigated the intraarticular administration of MSC from the aspects of the cartilage protective effect associated with their anti-inflammatory property and inhibitory effect on central sensitization of pain. When MIA was administered in this rat shoulder arthritis model, anti-Calcitonin Gene Related Peptide (CGRP) was expressed in the joint and C5 spinal dorsal horn. Moreover, expression of A disintegrin and metalloproteinase with thrombospondin motifs 5 (ADAMTS5), a marker of joint cartilage injury, was similarly elevated following MIA administration. When MSC were injected intraarticularly after MIA, the expression of CGRP in the spinal dorsal horn was significantly deceased, indicating suppression of the central sensitization of pain. The expression of ADAMTS 5 in joint cartilage was also significantly inhibited by MSC administration. In contrast, a significant increase in the expression of TNF-α stimulated gene/protein 6 (TSG-6), an anti-inflammatory and cartilage protective factor shown to be produced and secreted by MSC intraarticularly, was found to extend to the cartilage tissue following MSC administration. In this way, the intraarticular injection of MSC inhibited the central sensitization of pain and increased the expression of the anti-inflammatory and cartilage protective factor TSG-6. As the least invasive conservative strategies possible are desirable in the actual clinical setting, the intraarticular administration of MSC, which appears to be effective for the treatment of pain and cartilage protection in early-stage arthritis, may achieve these aims.

## 1. Introduction

The joint cartilage degeneration, injury and pain typically associated with arthropathy markedly impair patient quality of life (QOL). Thus, when considering treatment for affected joints, interventions that both help to preserve the joints themselves and also to control pain are required. In general, with regard to the pain aspect, noxious stimuli originating from the site of injury have been shown to be input into the spinal dorsal horn, with central sensitization then occurring [1,2,3]. A vicious cycle of pain results in which a secondary hypersensitivity to pain is induced [4,5,6,7]. Accordingly, when considering the treatment of arthropathy, in the same way as common therapeutic endpoints such as joint preservation and cartilage regeneration, it is also important to keep in mind issues such as pain control and central sensitization of pain [8].

In recent years, numerous investigations using mesenchymal stem cell (MSC) have been undertaken aiming to regenerate joint cartilage degenerated and injured during the course of osteoarthritis and other joint pathologies [9,10]. However, the results of cartilage regeneration obtained with intraarticular administration of MSC alone have not been encouraging, and surgical techniques using scaffolds are being increasingly attempted [11,12,13].

Most of these studies have focused on cartilage regeneration and proliferation, but studies from the viewpoint of the anti-inflammatory effect of MSC on the joint, pain control, and cartilage preservation are also needed, and may make possible non-invasive conservative therapy instead of surgical intervention whenever feasible. Also, while it is important to emphasize cartilage regeneration, as conservative therapy, attention must also be directed to prevention of arthropathy early in its course.

MSC also exert a potent anti-inflammatory effect, which has recently begun to be exploited in various fields [14,15]. Intraarticular inflammation is known to lead to further joint destruction. Accordingly, the pain-suppressing effect of MSC, which are considered to exert an anti-inflammatory action, and a preservation effect on the joint itself, namely their effects on prevention of disease progression as well as central sensitization of pain are worthy of further investigation.

This prompted us to prepare a shoulder arthritis model in the rat using monoiodoacetate (MIA) to provoke pain [16,17,18]. Using this model, the anti-inflammatory action of MSC administered intraarticularly was determined by Calcitonin Gene Related Peptide (CGRP) in the spinal dorsal horn by evaluating expression of TNF-α stimulated gene/protein 6 (TSG-6), an anti-inflammatory and cartilage preservation factor secreted by MSC, and central sensitization. In addition, the influence on the joint cartilage of the intraarticular administration of MSC was evaluated by quantifying the expression of the cartilage injury factor A disintegrin and metalloproteinase with thrombospondin motifs 5 (ADAMTS5).

## 2. Results

### 2.1. Immunofluorescence Staining of CGRP in the Spinal Dorsal Horn

**Figure 1:**
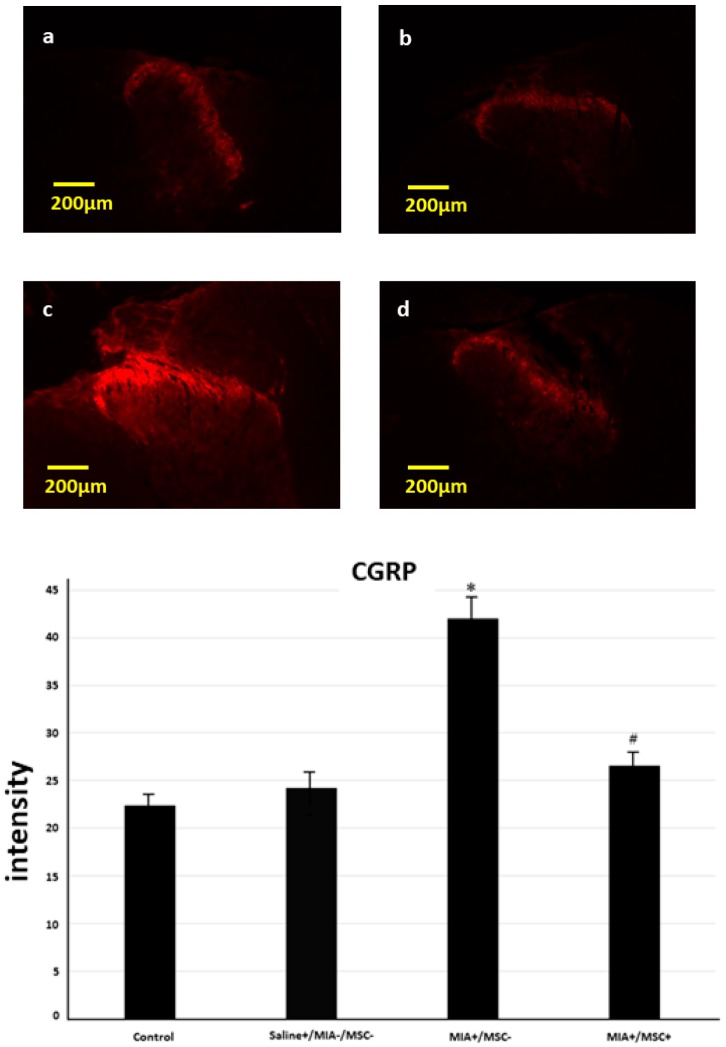
Calcitonin Gene Related Peptide (CGRP) expression in C5 spinal dorsal horn. (**a**) control group, (**b**) Saline group: CGRP is expressed in the posterior horn, (**c**) monoiodoacetate (MIA)+/mesenchymal stem cell (MSC)− group: CGRP is intensely expressed, (**d**) MIA+/MSC− group: CGRP expression as compared with MIA+/MSC− group is clearly decreased, and is suppressed to about the same degree in control and Saline group. In MIA+/MSC− group a significant increase is found, indicating that central sensitization has occurred. With MSC administration, as compared with MIA+/MSC− group a significant decrease is found meaning that central sensitization has been inhibited. * *p* < 0.001 compared to the untreated control. ^#^ *p* < 0.001 compared to the (MIA+/MSC−) group.

Since central sensitization of pain of shoulder joint origin can be investigated in the spinal dorsal horn of C5 [19,20], we studied CGRP expression at C5. The expression of CGRP at this site in the control group (untreated, Saline−/MIA−/MSC−) was compared with that of the other groups. Like the findings in the shoulder, expression in the Saline group spinal dorsal horn was virtually the same as that in the control. In contrast, in the (MIA+/MSC−) group C5 spinal dorsal horn significantly more intense CGRP expression was found. Meanwhile, in the (MIA+/MSC+) group CGRP expression as compared with MIA group was significantly decreased, and similar to that in the control and Saline groups. In this way, it could be confirmed that MSC administration inhibited central sensitization (Figure 1).

### 2.2. Histopathological Study

**Figure 2:**
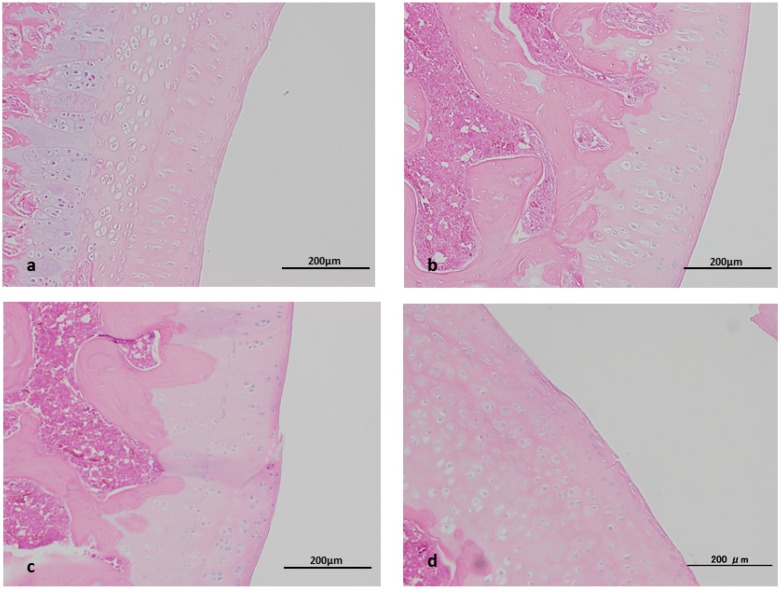
Histopathological study. (**a**) control group, (**b**) Saline group, (**c**) MIA+/MSC− group, (**d**) MIA+/MSC+ group. Control group and Saline group: Cartilage tissue is intact. MIA+/MSC− group: Although the cartilage tissue is preserved on the whole, the cartilage tissue surface layer shows partial injury. MIA+/MSC+ group: Although not as clearly as C, the cartilage tissue surface layer shows partial injury.

Histopathologically almost no differences were noted between the control and Saline groups. The transient pressure elevation induced by the injection of fluid was thus considered to have had no influence on the joint, confirming that simply changes induced by MIA and MSC would be recognized in this study. In the (MIA+/MSC−) group, partial injury of the joint and disappearance of the cartilage surface layer were found. In the (MIA+/MSC+) group partial injury was similarly present but none so severe as to lead to cartilage disappearance, while histopathologically no clear differences were apparent according to the presence/absence of MSC administration (Figure 2). We attribute this result to the fact that in this study the period of MSC administration was set at one week after MIA administration once intraarticular inflammation had already occurred. In this study, since changes in cartilage injury and cartilage protective factors were used to assess cartilage, when evaluation is impossible because the cartilage injury is diffusely severe or the cartilage has disappeared, the injection volume of MIA in this model was set at 0.3 mg and the evaluation period at 2 weeks, which were judged to be suitable parameters for the purposes of this study [16].

### 2.3. TSG-6 Expression in Joint Cartilage

**Figure 3:**
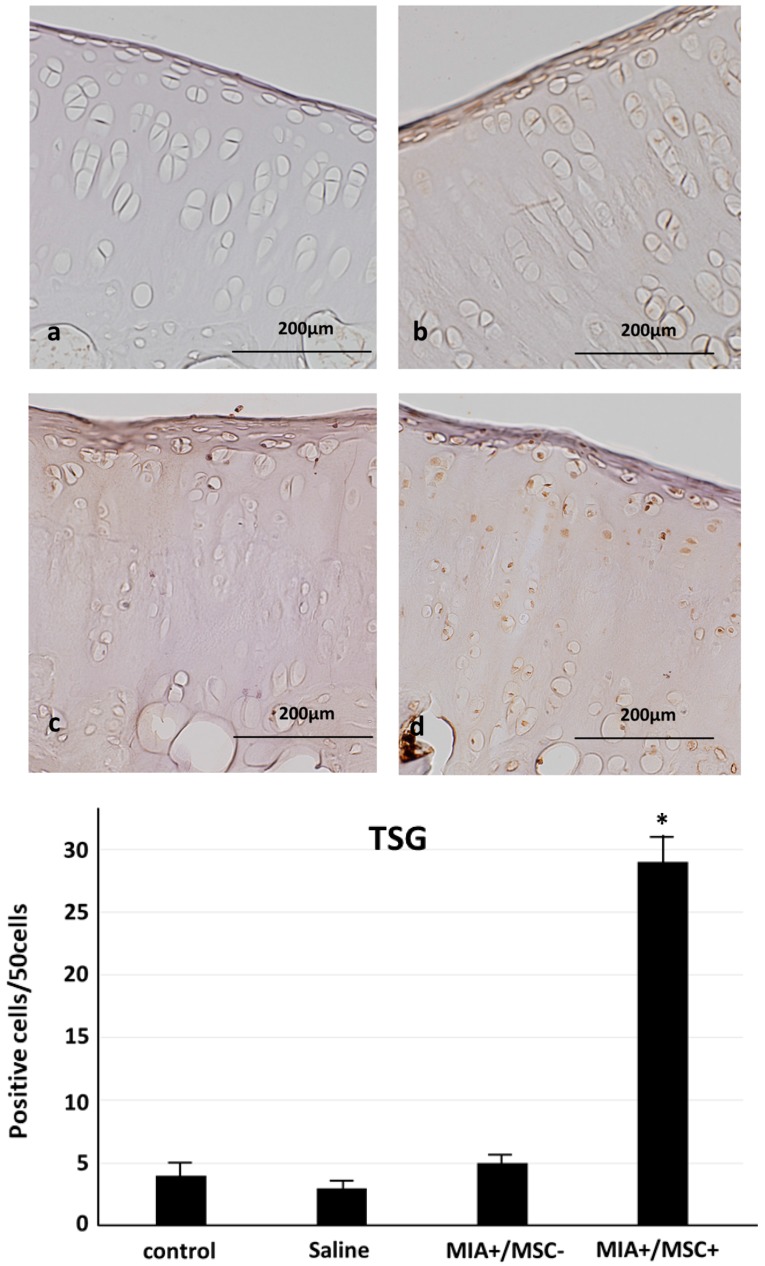
TNF-α stimulated gene/protein 6 (TSG-6) expression in cartilage tissue. (**a**) control group, (**b**) Saline group, (**c**) MIA+/MSC− group, (**d**) MIA+/MSC+ group. Control group and Saline group: Almost no TSG-6 expression is found in cartilage tissue. MIA+/MSC− group: Like in A, TSG-6 is not found anywhere in cartilage tissue. MIA+/MSC+ group: TSG-6 expression is found extensively from the surface layer to deep layer of cartilage tissue. As compared with control, Saline, MIA+/MSC− group, a significant increase in TSG-6 expression is found only in the group administered with MSC. * *p* < 0.01 compared to the MIA+/MSC− group.

The anti-inflammatory and cartilage protective factor TSG-6 which has been shown to be produced by MSC was studied. Almost no expression was found in the control group, saline group or MIA+/MSC− group. In contrast, TSG-6 which is known to be produced by MSC, showed a significant increase in expression in the cartilage tissue of MIA+/MSC+ group extending from the surface layer to deep layer. (Figure 3).

### 2.4. ADAMTS 5 Expression in Joint Cartilage

**Figure 4:**
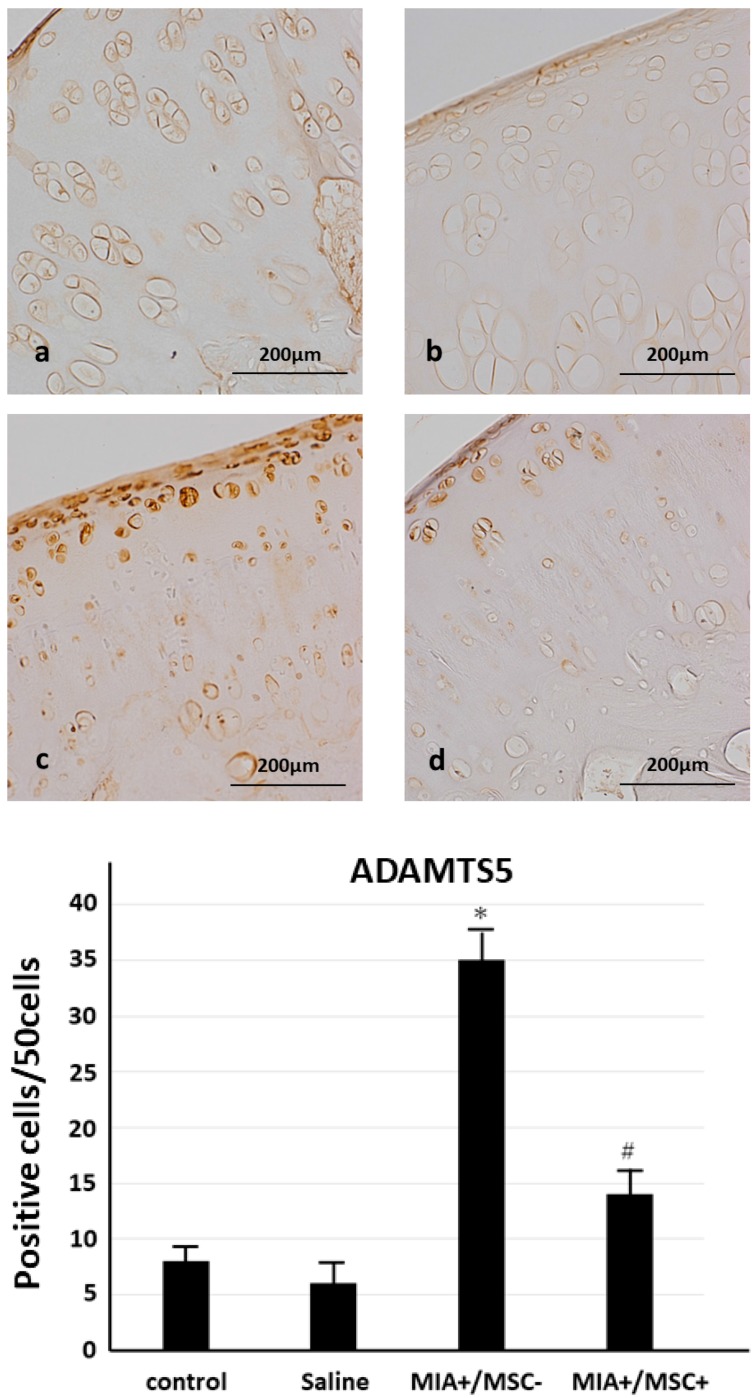
ADAMTS5 expression in cartilage tissue. (**a**) control group, (**b**) Saline group, (**c**) MIA+/MSC− group, (**d**) MIA+/MSC+ group. Control group and Saline group: Almost no ADAMTS5 is found in cartilage cells. MIA+/MSC− group: ADAMTS5 is extensively expressed from the hypertrophied cartilage layer to surface layer of cartilage cells. MIA+/MSC+ group: ADAMTS5 expression in cartilage cells is greater than in control and Saline group, but as compared with MIA+/MSC− group, ADAMTS5 expression is diffuse and clearly. A significant increase in ADAMTS5 expression is found in the MIA+/MSC− group. In the MIA+/MSC+ group as compared with A, B, it is increased, while as compared with the MIA+/MSC− group it is significantly decreased. * *p* < 0.001 compared to the untreated control. ^#^ *p* < 0.001 compared to the (MIA+/MSC−) group.

The expression of the cartilage injury factor ADAMTS5 was also studied. No clear differences were found between any of the groups in ADAMTS 5 expression in the joint cartilage surface layer, whereas in (MIA+/MSC−) group extremely intense ADAMTS5 expression was also noted in the joint cartilage deep layer. In (MIA+/MSC+) group scattered positive findings were seen in the deep layer, but significantly less so than in (MIA+/MSC−) group (Figure 4). These results suggest that the intraarticular injection of MSC may act to protect the joint and prevent osteoarthritis (OA) progression.

## 3. Discussion

When considering the treatment of arthropathy, it is important to keep in mind issues such as pain control and central sensitization of pain in the same way as more common therapeutic endpoints such as joint preservation and cartilage regeneration.

In general, noxious stimuli originating from the site of injury have been shown to be input into the spinal dorsal horn, with central sensitization then occurring [1,2,3]. This results in a vicious cycle of pain in which a secondary hypersensitivity to pain is induced. When considering the treatment of arthropathy, it is of course important to keep in mind the aspects of functional maintenance and pain control. This means that not only the condition within the joint, but also the state of central sensitization requires attention. Also, since intraarticular drug administration was used in the present study, we confirmed in the control and saline groups that no transient intraarticular pressure elevation was induced by injection of the drug solution. Namely, no changes in any parameter were induced by intraarticular saline injection in the untreated control group, meaning that transient pressure elevation did not exert any particular adverse effect on the joint or generation of pain. Accordingly, we judged that intraarticular drug injection as part of routinely performed therapy would not cause any particular problem.

MSC have been demonstrated to exert an anti-inflammatory action in a Bleomycin-induced lung injury model [21] and Lipopolysaccharide (LPS)-induced mouse peritonitis model [22]. Also, MSC produce the anti-inflammatory protein TSG-6, which has been reported to suppress the excessive inflammatory reaction occurring at ischemic sites in the myocardium, thereby exerting a myocardium-preserving effect [23]. In a rat cornea injury model as well, neutrophil infiltration, production of inflammation inducible cytokines, and development of corneal opacities have all been reported to be inhibited by the TSG-6 produced by MSC [24], documenting their potent anti-inflammatory action.

These findings drew our attention to the anti-inflammatory and pain suppressive properties of MSC, leading us to investigate the state of central sensitization after their intraarticular injection. Also, since shoulder joint pain evaluation in a shoulder joint disease model has been reported to be most feasible at C5, we focused on the C5 spinal dorsal horn [19,20]. As investigated items, pain and CGRP, which has been used in the evaluation of central sensitization in spinal dorsal horn inflammation [25,26,27], were applied. In addition, in the intraarticular study TSG-6, which is an anti-inflammatory factor secreted by MSC, was measured [23,24].

The results of this study revealed that the expression of CGRP in the C5 spinal dorsal horn was significantly increased in the MIA group as compared with the control and PS groups, confirming the occurrence of pain central sensitization. On the other hand, with MSC administration significant decreases to about the level of the control group were shown. In this way, the effect of MSC on pain control was demonstrated. With regard to TSG-6 expression in joint cartilage, it was confirmed to be significantly greater in MSC group. Accordingly, from the aspect of pain control taking into account both the local anti-inflammatory action and central sensitization, intraarticular injection of MSCs was considered to be effective. Moreover, TSG-6 itself has been reported to have a cartilage preserving effect [28], suggesting that intraarticular administration of MSC may also be directly advantageous for joint cartilage.

On the other hand, recently, various investigators have focused on the influence of MSC on cartilage regeneration and proliferation. When injected intraarticularly MSC scatter widely, making it impossible to obtain consistent local concentrations [29]. For this reason, hopes for their efficacy in enhancing cartilage regeneration and proliferation have been muted, and so instead surgical MSC implantation using scaffolds is being attempted. On the other hand, in cases in which the loss of joint cartilage is severe, namely in advanced osteoarthritis, surgery such as artificial joint replacement is commonly resorted to. Although in relatively young persons the postoperative results of artificial joints have been improving, their durability is still problematic, with multiple surgeries including revision often becoming necessary at comparatively young ages. With this in mind, the most conservative possible therapies need to be resorted to so as to gain time, with joint functional maintenance and prevention of cartilage injury together with pain control being the most important goals. Given this context rather than emphasizing joint cartilage regeneration and proliferation, the greater weight needs to be placed on prevention of arthropathic progression, for which reason our attention has been drawn to changes in joint cartilage preservation and injury factors.

In the present histopathological study, no obvious differences were noted between the MIA + MSC− and MIA + MSC+ groups. We attributed this result to the fact that we chose to study a period in which little histological injury had yet occurred because our intention was to characterize the joint preservation effect in early-stage disease, and because our second intention was to evaluate inflammation, and so to once induce inflammation we administered MIA to both groups and MSC one week later. In this study, ADAMTS5 was used as markers of joint cartilage degeneration and injury. Investigations using this factor were deemed appropriate because it has been reported that blockage of ADAMTS5 expression mitigates cartilage destruction [30], while resistance to OA in the ADAMTS5 knockout mouse has also been documented in multiple studies [31,32]. We found ADAMTS5 to be intensely expressed in joints administered MIA alone implicating this factor in the development of cartilage injury. In the group administered MSC ADAMTS5 expression was significantly inhibited, with this ascribed to a cartilage protective effect of MSC. Also, MSC characteristically exhibit homing at sites of injury and inflammation, and in the present study as well MSC migrated from sites of partial cartilage injury to within tissues, and by expressing TSG-6 showed changes thought to contribute to cartilage preservation.

Taking into consideration the results of other investigators as well, it can be understood that in OA so advanced that the joint cartilage has already disappeared the effect of intraarticular administration of MSC alone would be minimal. However, given that in this study joint preservation factors were expressed in joint cartilage, an adequate effect could well be expected from the intraarticular administration of MSC at an early disease stage, namely, when cartilage injury is still mild and the morphology of cartilage tissue still preserved.

The results of this study suggest that the intraarticular administration of MSC in early-stage arthritis and OA may, at least at this stage, be beneficial in improving inflammation, decreasing central sensitization and preserving cartilage.

## 4. Materials and Methods

### 4.1. Animals

Male Sprague-Dawley rats aged 10 weeks and weighing 220 to 250 g (Sankyo Labo Service, Tokyo, Japan) were used. All rats were housed under standard laboratory conditions (temperature 24 °C, 12 h light/dark cycle) and were given food and water ad libitum. This study was conducted in accordance with all guidelines of the Animal Research Committee of Kanazawa Medical University (#2017-45; approval date: 1 April 2017).

### 4.2. Cell Culture

Rat mesenchymal stem cells (Cyagen, DS Pharma Biomedical, Tokyo, Japan) were maintained as a subconfluent monolayer culture in Mesenchymal Stem Cell Growth Medium (Cyagen, DS Pharma Biomedical) at 37 °C under 5% CO_2_/95% air. After the culture reached 80% confluency the cells were harvested by trypsinization. Then rats were injected with rat mesenchymal stem cells 5.0 × 10^6^ cells into the left shoulder.

### 4.3. Treatment and Tissue Preparation

The animals were randomized and grouped (𝑛 = 8 per group) before the initiation of the study. All rats were anesthetized with an intraperitoneal (i.p.) injection of sodium pentobarbital and treated aseptically. Their left shoulders were treated with a single intraarticular injection of 0.3 mg of MIA (Sigma-Aldrich, St. Louis, MO, USA). In addition, to detect any intraarticular changes induced by transient elevations of intraarticular pressure due to saline injection, the left shoulders of the untreated control group were evaluated. The rats were then randomly divided into four groups: An untreated control group (no injection), (Saline+/MIA−/MSC−) group (injected saline, no MIA injection), (MIA+/Saline+) group (one week after MIA injection intraarticular injection of saline), (MIA+/MSC−) group (one week after MIA injection intraarticular injection of 5.0 × 10^6^ MSC). On the 14th day after the start of the experiment the spinal cord at C5 and left shoulder joint were extracted in all groups. Rat spinal dorsal horns at C5 were frozen and embedded in OCT compound (Sankyo Co., Tokyo, Japan) for immunofluorescence imaging of Calcitonin Gene Related Peptide (CGRP). Rat shoulder joints were fixed in 10% neutral buffered formalin, and embedded in paraffin for hematoxylin and eosin staining and immunostaining of ADAMTS 5 and TSG-6.

### 4.4. Histopathology of the Shoulder Joint

The specimens were continuously demineralized in 10% EDTA followed by standard histological techniques using paraffin blocks. The samples were serially sectioned in steps of 4 μm, stained using H–E, and assessed by light microscopy.

### 4.5. Immunofluorescence Imaging

To evaluate central sensitization in the spinal dorsal horn, the state of calcitonin-gene-related peptide (CGRP) expression in the spinal dorsal horn was determined immunohistochemically. The frozen tissue sections (10 μm) were cut using a cryostat (CM1950, Leica microsysteme, Leica, Germany). These sections were stained with affinity-purified anti-Calcitonin Gene Related Peptide (CGRP) rabbit polyclonal antibody (Calbiochem, La Jolla, CA, USA) at a concentration of 1.0 μg/mL, antibody for 2 h followed by a fluorescent-labeled secondary antibody (Alexa 594, Invitrogen, Eugene, OR, USA) for 30 min. Nonspecific binding was blocked by incubating sections with 10% goat serum (DakoCytomation, Carpinteria, CA, USA) in PBS containing 0.02% Triton X-100 for 15 min. After washing, Prolong Diamond antifade Mountant (Invitrogen) was added and cover slips were mounted. Images were acquired using the Zeiss-LSM710 (Baden-Württemberg, Oberkochen, Germany). Then using the LSM software ZEN 2010 (CarlZeiss, Oberkochen, Germany), the intensity in all of the groups was measured and compared.

### 4.6. Immunostaining for ADAMTs 5, Stimulated Gene 6 Protein (TSG-6)

To confirm the anti-inflammatory effect of MSC in the shoulder joint using anti-TSG6 antibody, expression of each factor in the joint cartilage was evaluated immunohistochemically using anti-ADAMTS5 antibody in the cartilage as a marker of shoulder cartilage injury. To examine the expression of ADAMTS5 and TSG-6 in cartilage, the cartilage was stained immunohistochemically with anti-ADAMTS5 antibody and anti-TSG6 antibody. Briefly, tissue sections were thoroughly deparaffinized and rehydrated. For antigen retrieval, the sections were dipped into the protease K at 37 °C for 30 min. After washing in PBS, endogenous peroxidase activity was inhibited with normal goat or rabbit serum (Nichirei Co., Tokyo, Japan) for 30 min. Tissue sections were incubated overnight at 4 °C with affinity-purified anti-ADAMTs5 or anti-TSG6 rabbit polyclonal antibodies (Funakoshi, Tokyo, Japan), (abcam204049, Tokyo, Japan) at a concentration of 10 μg/mL each. The sections were incubated with 10 g/mL biotinylated rabbit anti-mouse immunoglobulin (Nichirei Co.) for 30 min. After washing in PBS, they were incubated with 100 mg/mL horseradish peroxidase (HRP)-conjugated streptavidin (Nichirei Co.) for 30 min. The color reaction was performed with 0.05 Tris-HCl (pH 7.6) containing 3,3′-diaminobendizine tetrahydrochloride (Nichirei Co.) and the sections were counterstained with Meyer’s hematoxylin. A BX53 microscope (Olympus, Tokyo, Japan) was used. The camera used was DP71 (Olympus). Like in previous reports, the number of positive cells in each group was calculated as described below, and the differences subjected to statistical analysis [33]. The number of positively stained cells was counted in articular cartilage. Each section was observed under a light microscope at 400× *g* magnification. The mean number of positive cells/50 cells was calculated in each group. All slides were evaluated independently by 2 blinded observers (Toru Ichiseki and Miyako Shimasaki).

### 4.7. Statistical Analysis

All data are presented as mean ± SE. Differences between groups were assessed using the one-way analysis of variance followed by Fisher’s protected least significant difference post hoc test. Differences were considered significant at *p* < 0.05.

## 5. Conclusions

The intraarticular administration of MSC was demonstrated to inhibit central sensitization of pain, and based on measurements of TSG-6 and ADAMTS5 in joint cartilage to help preserve it. These results suggest that intraarticular MSC administration, a minimally invasive conservative technique, may be a promising therapeutic option in lieu of surgical intervention to preserve cartilage in early arthropathy.

## Abbreviations

MIA	Monoiodoacetate
MSC	Mesenchymal stem cell
ADAMTS5	A disintegrin and metalloproteinase with thrombospondin motifs 5
CGRP	Calcitonin gene related peptide
TSG-6	TNF-α stimulated gene/protein 6
OA	Osteoarthritis
